# Phylogenetic and Molecular Characterization of a Novel Reassortant High-Pathogenicity Avian Influenza A (H7N6) Virus Detected in New Zealand Poultry

**DOI:** 10.3390/ijms262110623

**Published:** 2025-10-31

**Authors:** Andrew Wilson, Ruy Jauregui, Edna Gias, Yee Syuen Low, Alvey Little, Helen Johnston, Wlodek Stanislawek, Anastasia Chernyavtseva, Michelle McCulley

**Affiliations:** Animal Health Laboratory, Ministry for Primary Industries, 66 Ward St, Upper Hutt, Wellington 5018, New Zealand; andrew.wilson2@mpi.govt.nz (A.W.); ruy.jauregui@mpi.govt.nz (R.J.); edna.gias@mpi.govt.nz (E.G.); yeesyuen.low@mpi.govt.nz (Y.S.L.); alvey.little@mpi.govt.nz (A.L.); helen.johnston@mpi.govt.nz (H.J.); wlodek.stanislawek@mpi.govt.nz (W.S.); anastasia.chernyavtseva@mpi.govt.nz (A.C.)

**Keywords:** avian influenza virus, HPAI, genomics, H7N6, poultry, reassortant, New Zealand

## Abstract

H7 high-pathogenicity avian influenza (HPAI) virus outbreaks can cause high rates of morbidity and mortality in poultry flocks, leading to devastating impacts on poultry industries. In December 2024, an HPAI virus was detected on a poultry farm in New Zealand, being the first time a case of HPAI was reported in the country. Whole-genome sequencing, subtyping, phylogenetic, and mutation analyses were performed to characterize the virus. Results indicated a novel high-pathogenicity H7N6 avian influenza virus arose through a reassortment event between endemic low-pathogenicity H4N6 and H7 viruses, followed by two mutations at the H7 gene cleavage site. Mutation analysis suggests the novel H7N6 virus exhibits increased risk of host specificity shift, but further work is required to fully understand the functional impacts of the detected mutational events. In this instance, a timely biosecurity response was effective in eliminating the virus and preventing its transmission to secondary poultry flocks in New Zealand. However, the event underscores the critical importance of continued surveillance of commercial poultry and other potential avian carriers to facilitate early detection of low-pathogenicity avian influenza viruses, which may undergo reassortment or de novo mutation into high-pathogenicity variants.

## 1. Introduction

Avian influenza viruses (AIVs) are pathogens belonging to the genus *Alphainfluenzavirus* of the family *Orthomyxoviridae* and are responsible for avian influenza (commonly known as bird flu), a disease with significant global economic impact [1]. AIVs are named according to the combination of one of 16 haemagglutinin (HA) gene subtypes, and one of 9 neuraminidase (NA) subtypes present in the viral genome [2]. AIVs are classified as either high- (HPAI) or low-pathogenicity (LPAI) based on HA gene features [3]. HPAI viruses possess a polybasic HA cleavage site, enabling systemic infection [3,4]. In comparison, LPAI viruses feature a monobasic HA cleavage site, often restricting replication to respiratory and intestinal tissues [3,5]. Whilst most AIVs are classified as LPAI, only viruses with HA subtypes H5 or H7 demonstrate the potential to evolve into HPAI, which can cause severe disease and mortality in wild and domestic birds [6]. In addition, HPAIs can gain the ability to infect new hosts, such as ruminants, felines, pinnipeds, and primates, including humans, leading to major impacts on agriculture, trade, and public health [7]. For AIVs to infect humans, mutational events within the HA receptor binding site sequence are required to change sialic acid receptor binding preference from α2,3-linked (avian-like) to α2,6-linked (human-like) [8]. Located in the distal head of the HA1 protein subunit, the receptor binding site includes three conserved motifs, the 130- and 220-loops, and the 190-helix, alongside key amino acid motifs that determine sialic acid receptor binding preference [9]. Therefore, molecular characterization of both the HA cleavage and receptor binding sites are fundamental in understanding pathogenicity and potential host range [10,11].

AIVs feature a negative sense, single-stranded, RNA genome consisting of eight segments that encode various viral proteins [12]. Given the segmented nature of AIV genomes, reassortment between circulating strains of AIV can give rise to viruses with novel phenotypic and clinical characteristics, such as increased virulence and higher zoonotic potential [13,14]. Of increasing international interest are H5N1 HPAI viruses belonging to subclade 2.3.4.4b, which cause significant outbreaks in avian populations, with specific genomic mutations such as PB2-E627K enhancing its capacity for mammalian adaptation and human infection [15,16]. Given the high risk to both animal and human health, the World Organization for Animal Health (WOAH) classifies HPAI as a global pathogen of concern [17].

Wild waterfowl serve as natural reservoirs for AIVs, and migratory movements facilitate both local and long-distance transmission of LPAI and HPAI strains [18,19,20]. Research programs focusing on the prevalence of AIVs in mallard ducks (*Anas platyrhynchos*) dating back to the 1980s have described the endemic populations of LPAIs in New Zealand, with the most frequently isolated strains characterized as H4N6, H3N8, and H10N3 [21,22]. Given its geographical isolation and distant proximity to the East Asian–Australasian migratory flyway, New Zealand had long maintained freedom status from HPAI [21]. However, in late 2024, New Zealand’s first documented case of H7 HPAI, designated A/Chicken/NZ/W24_2595/2024 (H7N6), was detected on a commercial free-range poultry farm on the South Island [23].

This study systematically investigates the phylogenetic and molecular characteristics of New Zealand’s novel H7N6 HPAI in relation to both international and domestic AIVs. The detection and characterization of the first domestic case of H7 HPAI marks a significant epidemiological event for New Zealand and emphasizes the importance of continued nationwide AIV surveillance.

## 2. Results

### 2.1. Genomic Analysis of Global H7 Viruses Indicates a Pathogenic Shift from a Domestic Strain of H7 LPAI

**Figure 1:**
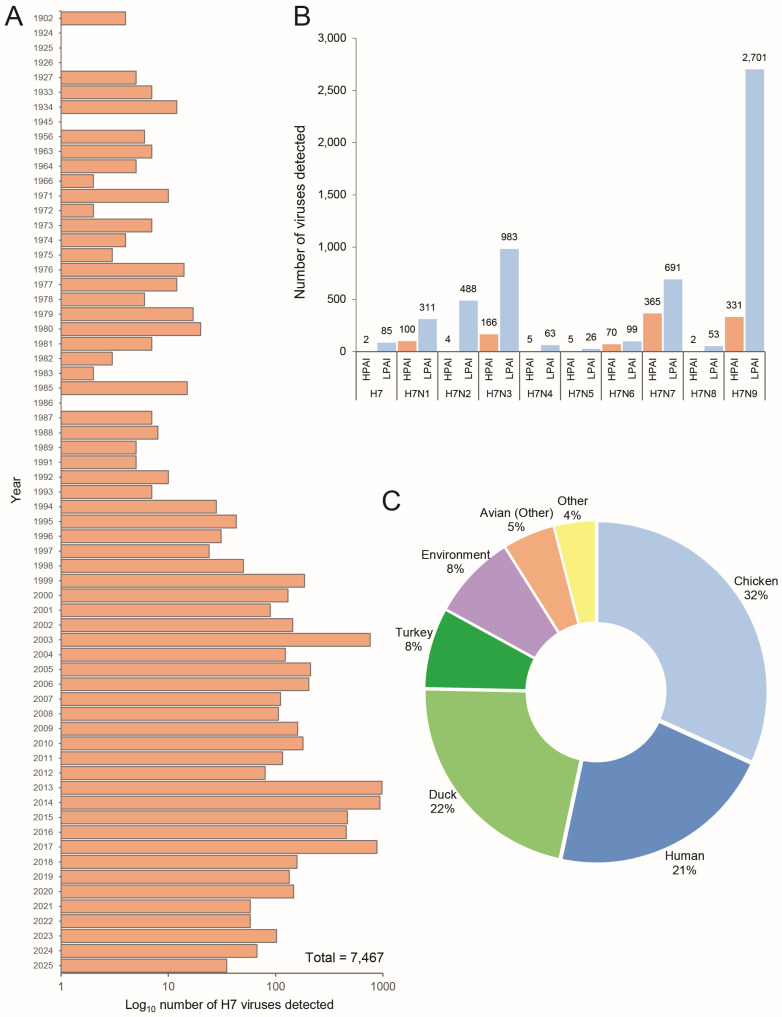
Distribution of H7 influenza viruses available in the GISAID EpiFlu™ database. (**A**) Temporal distribution of H7 viruses characterized and uploaded to the GISAID EpiFlu™ database (Log_10_ transformed). (**B**) Number of H7 HPAI vs. LPAI genome sequences available in the GISAID EpiFlu™ database, sorted by subtype. (**C**) Host species distribution for H7 viruses.

**Figure 2:**
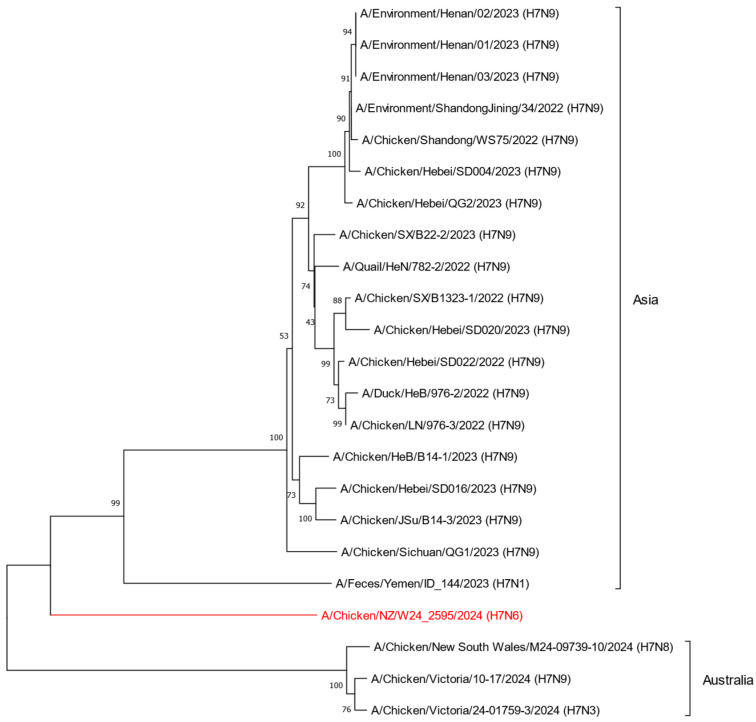
Phylogenetic placement of the New Zealand H7N6 HPAI virus in relation to other recently detected H7 HPAI viruses from Asia and Australia. The phylogenetic tree depicts the HA segment. All genomes were obtained from the GISAID EpiFlu™ database. The New Zealand H7N6 HPAI HA segment is outlined in red. Evolutionary history was inferred using the Maximum Likelihood method. Evolutionary distances were computed using the Tamura-Nei (1993) method allowing for evolutionary invariability. Evolutionary analyses were conducted in MEGA11 [25].

The first recorded outbreak of H7 AIV in chickens occurred in Italy in 1902, and since then, H7 AIVs have been circulating globally in various host species [24]. To understand the global distribution of H7 influenza viruses, we obtained and analyzed all available metadata in the GISAID EpiFlu™ database (n = 7647). Although first detected in 1902, cases of H7 AIVs began to drastically increase across the globe in the late 1990s (Figure 1A). Of all documented H7 AIVs in the GISAID EpiFlu™ database, the majority of H7 AIVs are characterized as H7N9 (n = 3032), with over 89% (n = 2701) of those further characterized as LPAI (Figure 1B). Further to this, H7 viruses hold the potential to infect a wide range of host species (Figure 1C). Of note, chickens, ducks, and humans were the most well represented host groups in the EpiFlu™ database (32%, 22%, and 21%, respectively), followed by turkeys (8%), environmental detections (8%), and other avian species (5%). Given that H7 viruses are well represented in the GISAID EpiFlu™ database, we obtained genome sequences for H7 HPAI viruses detected between 2022 and 2024 in Asia and Oceania to determine the phylogenetic placement of New Zealand’s H7N6 HPAI in relation to international strains. Phylogenetic analysis shows that the H7N6 HPAI is genetically distinct from other H7 HPAI viruses previously detected across Asia and Oceania (Figure 2). Analysis of the H7N6 HPAI HA gene sequence using NCBI BLAST yielded a 94.7% pairwise identity top hit to a domestic H7N7 LPAI virus detected in 2017 (GenBank ID: PX271007.1). This finding strongly indicated a shift in pathogenicity from a domestic strain of H7 LPAI.

During the New Zealand HPAI biosecurity response in December 2024, we detected and characterized an H4N6 LPAI virus in a sample collected from a wild mallard duck found approximately 30 kilometres south of the H7N6 HPAI index property. All eight segments of the AIV genome were enriched from the positive sample using a RT-PCR targeting the conserved regions of each genome segment, followed by whole-genome sequencing and subtype assignation. The virus was designated A/Mallard/NZ/W24_2774/2024 (H4N6), with genome organization consistent with other avian influenza viruses (Table 1). Initial NCBI BLASTn analyses revealed the NA segment featured a remarkable degree of similarity (98.3% pairwise identity) to the H7N6 HPAI virus. Given this finding, we hypothesized that the H4N6 virus detected near the HPAI index property may represent one of the precursor viruses that reassorted with an endemic H7 LPAI virus prior to a shift in pathogenicity.

### 2.2. Phylogenetic Analysis of the H7N6 HPAI Virus Provides Strong Evidence of Reassortment Between Endemic LPAI Viruses

**Figure 3:**
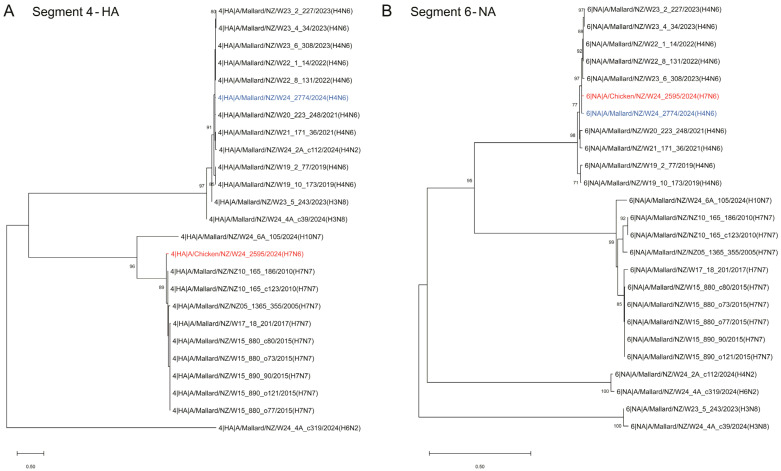
Phylogenetic analysis of HA and NA genes for the H7N6 HPAI and H4N6 LPAI viruses. Phylogenetic trees depicting (**A**) HA and (**B**) NA segments. Sequences were generated as part of an internal avian influenza genome database. H7N6 HPAI HA and NA segments are outlined in red. H4N6 LPAI HA and NA segments are outlined in blue. Evolutionary history was inferred using the Maximum Likelihood method. Evolutionary distances were computed using the Tamura-Nei (1993) method allowing for evolutionary invariability. Evolutionary analyses were conducted in MEGA11 [25].

To further investigate the notion of a reassortant virus, phylogenetic analysis was performed for each of the eight gene segments in relation to both endemic LPAI strains of H7N7 and H4N6. First, phylogenetic trees were constructed using the full-length nucleotide sequences of both the HA and NA genes, which provided support for classification as H7N6, and demonstrated a strong evolutionary relationship between the H7N6 HPAI virus and domestic strains of AIV (Figure 3). This evidence supports that the H7N6 HPAI virus arose onshore in a de novo manner. Furthermore, phylogenetic analysis of the HA gene revealed a close evolutionary link to various H7N7 LPAI sequences isolated in New Zealand (Figure 3A), and analysis of the NA gene provided evidence of a genetic relationship between the H7N6 HPAI and H4N6 LPAI sequences (Figure 3B). The close phylogenetic placements to endemic H7N7 LPAI (HA gene) and H4N6 LPAI (NA gene) sequences suggest these strains were the precursor viruses prior to reassortment into the H7N6 HPAI.

**Figure A1:**
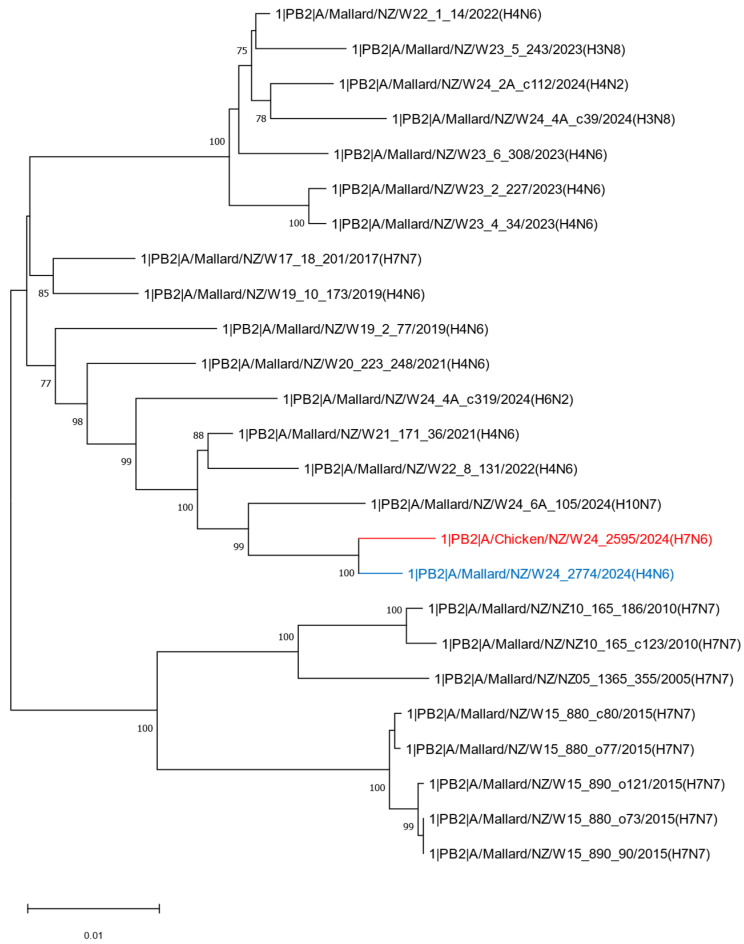
Phylogenetic analysis of the PB2 gene for the H7N6 HPAI and H4N6 LPAI viruses. H7N6 HPAI PB2 sequence is outlined in red. H4N6 LPAI PB2 sequence is outlined in blue. Sequences outlined in black are part of an internal avian influenza genome database. Evolutionary history was inferred using the Maximum Likelihood method. Evolutionary distances were computed using the Tamura-Nei (1993) method allowing for evolutionary invariability. Evolutionary analyses were conducted in MEGA11 [25].

**Figure A2:**
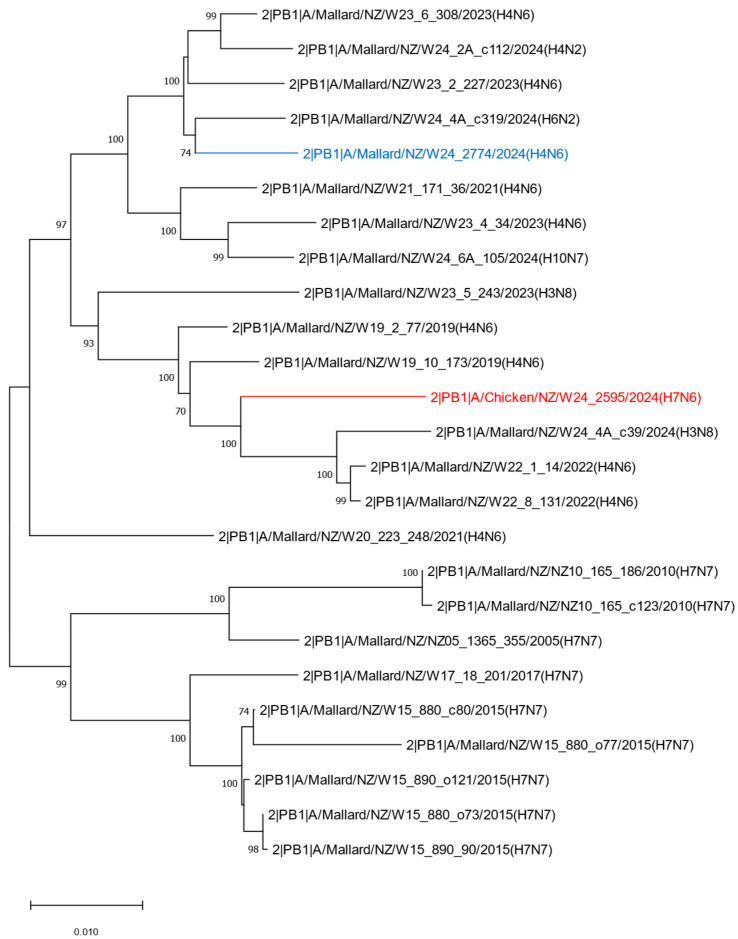
Phylogenetic analysis of the PB1 gene for the H7N6 HPAI and H4N6 LPAI viruses. H7N6 HPAI PB1 sequence is outlined in red. H4N6 LPAI PB1 sequence is outlined in blue. Sequences outlined in black are part of an internal avian influenza genome database. Evolutionary history was inferred using the Maximum Likelihood method. Evolutionary distances were computed using the Tamura-Nei (1993) method allowing for evolutionary invariability. Evolutionary analyses were conducted in MEGA11 [25].

**Figure A3:**
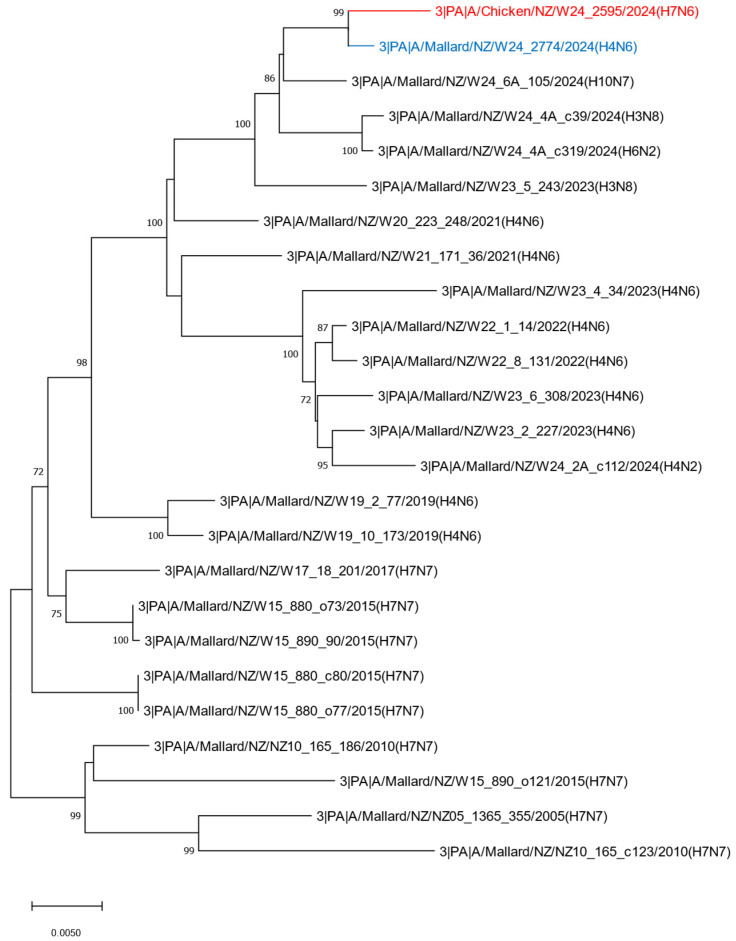
Phylogenetic analysis of the PA gene for the H7N6 HPAI and H4N6 LPAI viruses. H7N6 HPAI PA sequence is outlined in red. H4N6 LPAI PA sequence is outlined in blue. Sequences outlined in black are part of an internal avian influenza genome database. Evolutionary history was inferred using the Maximum Likelihood method. Evolutionary distances were computed using the Tamura-Nei (1993) method allowing for evolutionary invariability. Evolutionary analyses were conducted in MEGA11 [25].

**Figure A4:**
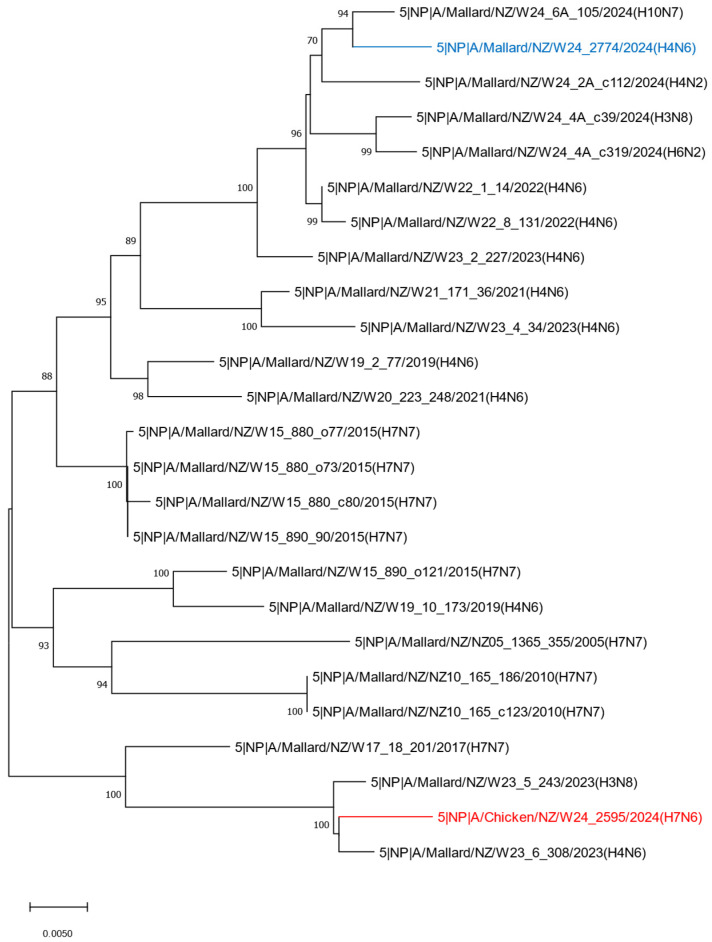
Phylogenetic analysis of the NP gene for the H7N6 HPAI and H4N6 LPAI viruses. H7N6 HPAI NP sequence is outlined in red. H4N6 LPAI NP sequence is outlined in blue. Sequences outlined in black are part of an internal avian influenza genome database. Evolutionary history was inferred using the Maximum Likelihood method. Evolutionary distances were computed using the Tamura-Nei (1993) method allowing for evolutionary invariability. Evolutionary analyses were conducted in MEGA11 [25].

**Figure A5:**
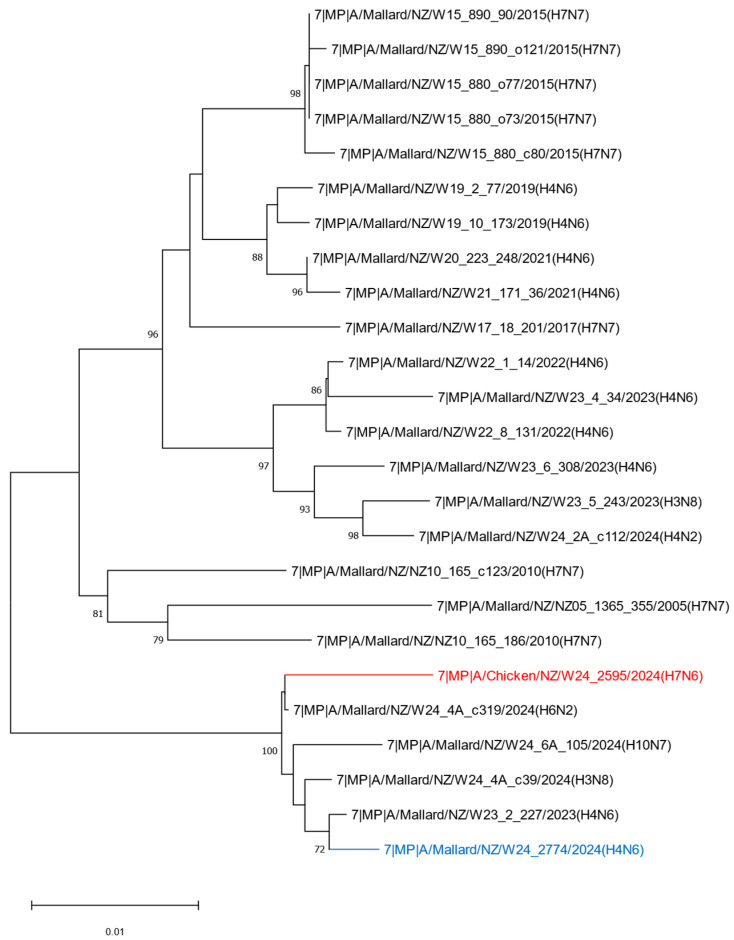
Phylogenetic analysis of the MP gene for the H7N6 HPAI and H4N6 LPAI viruses. H7N6 HPAI MP sequence is outlined in red. H4N6 LPAI MP sequence is outlined in blue. Sequences outlined in black are part of an internal avian influenza genome database. Evolutionary history was inferred using the Maximum Likelihood method. Evolutionary distances were computed using the Tamura-Nei (1993) method allowing for evolutionary invariability. Evolutionary analyses were conducted in MEGA11 [25].

**Figure A6:**
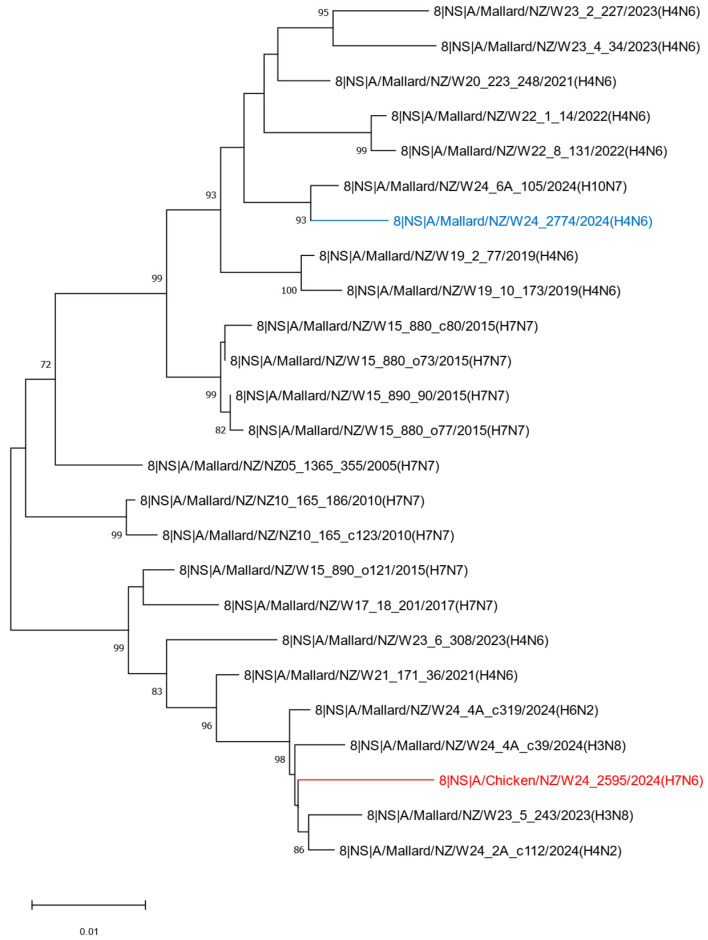
Phylogenetic analysis of the NS gene for the H7N6 HPAI and H4N6 LPAI viruses. H7N6 HPAI NS sequence is outlined in red. H4N6 LPAI NS sequence is outlined in blue. Sequences outlined in black are part of an internal avian influenza genome database. Evolutionary history was inferred using the Maximum Likelihood method. Evolutionary distances were computed using the Tamura-Nei (1993) method allowing for evolutionary invariability. Evolutionary analyses were conducted in MEGA11 [25].

To further understand the evolutionary history of the H7N6 HPAI virus, phylogenetic analyses were performed on the remaining six internal genes: PB2, PB1, PA, NP, MP, and NS (Appendix A, Figure A1, Figure A2, Figure A3, Figure A4, Figure A5 and Figure A6). Of note, the PB2 and PA genes detected in the H7N6 HPAI virus demonstrate a close evolutionary relationship to the PB2 and PA genes derived from the H4N6 LPAI genome detected near the site of HPAI infection, further supporting the notion of a reassortant strain. The remaining internal genes, PB1, NP, MP, and NS, more closely aligned to other endemic strains of AIV previously detected in New Zealand and are likely derived from the precursor H7 LPAI virus that remains uncharacterized.

**Figure 4:**
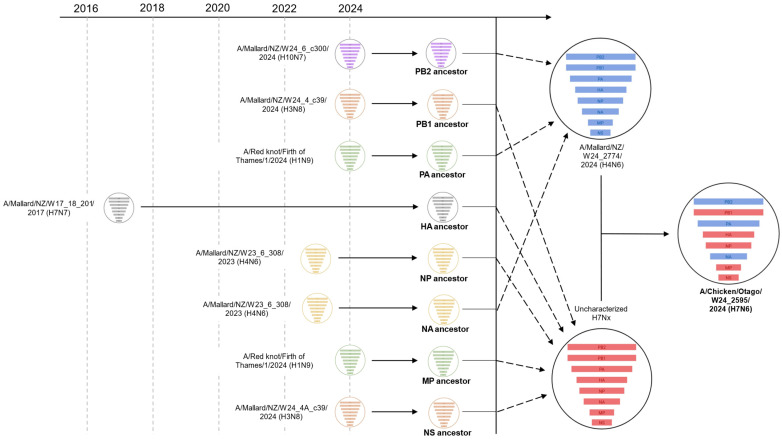
Predicted mode of reassortment and closest viral ancestors of each H7N6 HPAI genome segment. The most recent genome with the highest BLAST pairwise identity was selected as a candidate for the closest viral ancestor for a particular segment.

Given that we were unable to characterize the precursor H7 LPAI virus that is hypothesized to have reassorted with the H4N6 LPAI strain, we performed additional NCBI BLAST analyses for each full-length H7N6 HPAI gene sequence (Table 2). As expected, high homology to A/Mallard/NZ/W24_2774/2024 (H4N6) was observed for PB2, PA, and NA gene sequences (99.1%, 99.2%, and 98.8% pairwise identity, respectively), whereas the remaining gene sequences, PB1, HA, NP, MP, and NS, align closer to a range of other endemic strains of AIV that share recent common ancestry of the uncharacterized H7 precursor virus (Table 2). Figure 4 summarizes the predicted mode of reassortment between domestic strains of AIVs, culminating in precursor H4N6 and uncharacterized H7 genomes that, upon reassortment and mutation of the HA cleavage site, resulted in the H7N6 HPAI virus.

### 2.3. Molecular Analysis of the H7N6 HPAI Virus Reveals Mutational Events Associated with Changes in Host Specificity and Host Cell Receptor Binding

To assess the risk of potential zoonotic spillover into mammalian species, a thorough mutational analysis for each H7N6 HPAI gene was performed. As previously reported by McCulley et al. 2025, the H7N6 HPAI cleavage site between the HA1 and HA2 proteins features the polybasic amino acid motif PEGPKRRKR/GLF, caused by the combination of a 6 bp insertion and a single nucleotide variant in the nucleotide sequence directly preceding the cleavage site [23].

Mutation analysis of each gene segment was performed using the GISAID FluSurver tool and are presented in Supplementary Table S1. Of the significant mutational events observed, five mutations were predicted to be associated with host specificity shift and/or host cell receptor binding, and four predicted to affect virulence (Table 3). PB2-I292V is an adaptive marker documented to promote replication in mammalian hosts and has been detected in emergent H7 viruses isolated from humans [26]. For influenza viruses with the PB2-I292V mutation, in vivo studies have demonstrated increased replication and greater pathogenic potential through the enhancement of viral polymerase activity and attenuation of interferon-β induction [26]. However, the PB2 mutation most associated with mammalian adaptation, PB2-E627K, was not detected.

**Figure 5:**
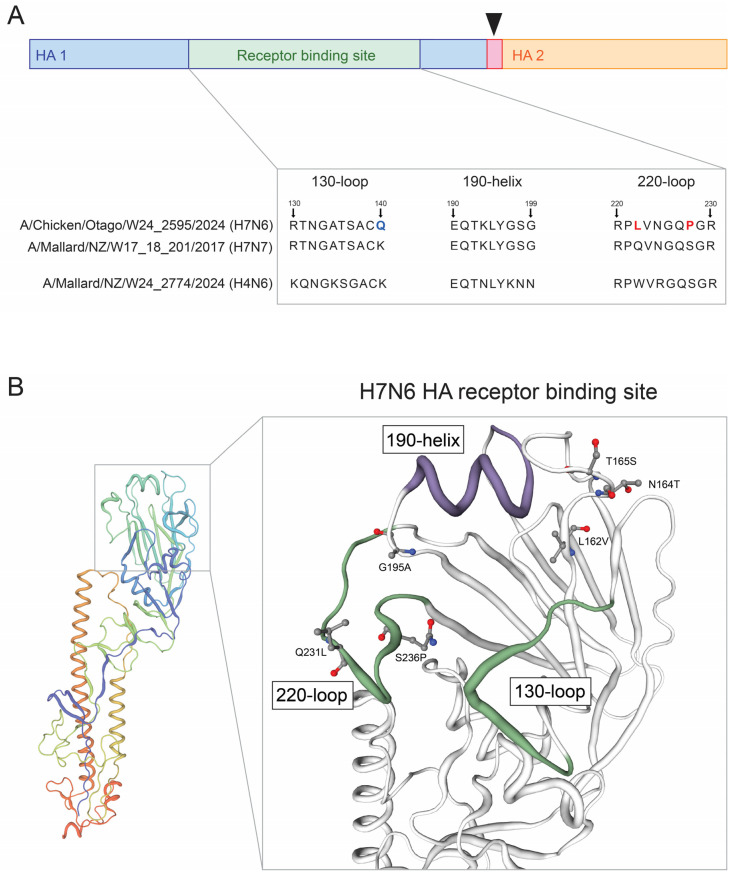
Protein structure modelling reveals mutations within the receptor binding site of the H7N6 HPAI haemagglutinin gene. (**A**) schematic of the HA receptor binding site and the amino acid sequences of the 130-loop, 190-helix, and 220-loop domains. The HA cleavage site is marked by the black triangle. Mutational events called using the FluSurver tool are marked in red. Mutational events called through comparison to a New Zealand H7N7 LPAI genome are marked in blue. (**B**) protein structure modelling of the H7N6 HPAI HA receptor binding site, annotating the six HA receptor binding site mutations in relative proximity to the key loop and helix domains. The 130-loop and 220-loop domains are coloured green. The 190-helix domain is coloured purple. Protein structure modelling was performed using the SWISS-MODEL workspace [36].

Genomic analysis revealed six single nucleotide mutational events within the H7N6 HPAI HA gene that are predicted to affect host specificity and host cell receptor binding, or affect the HA receptor binding site: HA-L162V, -N164T, -T165S, -G195A, -Q231L, and -S236P (annotated using H7 numbering). Given the potential functional significance of these mutations, we first analyzed the receptor binding site of the H7N6 HPAI HA protein to investigate sialic acid (SA) binding specificity. The HA receptor binding site features the residues E190, G225, Q226, and G228, which are associated with α2,3-linked SA binding specificity in H7 viruses (avian-like), and were found to be conserved after comparison to H7N7 LPAI, and H4N6 LPAI HA genes (Figure 5A) [9]. Whilst the 130-loop domain for the H7N6 HPAI features the K140Q mutation when compared to the H7N7 LPAI sequence (marked blue in Figure 5A), this mutational event was not called using FluSurver as the reference sequence used for analysis also presents with a Q at residue 140. Sites of each of the six mutational events affecting the HA receptor binding site, and their relative proximities to the key functional domains of the receptor binding site, are shown in Figure 5B. Finally, two further mutational events were called in the H7N6 HPAI genome that are predicted to remove potential N-glycosylation sites, HA-T143A and NA-N54S, which may impact viral infectivity, stability, protein structure, and the resulting host immune response (Supplementary Table S1) [35].

Overall, the H7N6 HPAI virus features several mutations that are predicted to increase virulence and promote host susceptibility shifts. However, structural proteomic studies are required to fully elucidate the functional impacts of these mutations, which are outside the scope of this work.

## 3. Discussion

This study comprehensively characterized the phylogenetic and molecular characteristics of New Zealand’s first case of H7 HPAI that was detected on a free-range poultry farm in late 2024 [23]. Given the vast amount of sequence data available for H7 influenzas in the GISAID EpiFlu™ database, we were able to confidently exclude the possibility of an HPAI incursion from overseas through phylogenetic analysis of HA gene sequences. Further phylogenetic analysis including domestic H7N7 LPAI viruses provided evidence of an onshore pathogenicity switch from LPAI to HPAI. Spontaneous mutational events that lead to a change in pathogenicity have been reported globally, often following prolonged circulation in domestic poultry populations that interact with wildlife [37,38,39,40]. Whilst serological testing has found no evidence of active AIV infections in New Zealand domestic poultry, the detection of an HPAI virus on a free-range commercial poultry farm in New Zealand was expected due to both direct and indirect contact with nearby waterfowl [21]. In addition, we were able to characterize a H4N6 LPAI virus detected in a wild mallard duck near the infected free-range poultry farm. Genomic analysis of the H4N6 virus revealed high similarity to three of the eight H7N6 HPAI gene sequences, leading to our hypothesis that the H7N6 HPAI was a reassortant strain. Further phylogenetic and evolutionary analysis provided evidence supporting this hypothesis, indicating a reassortment event between an H4N6 and an uncharacterized domestic H7 virus.

Molecular characterization of the H7N6 HPAI virus HA receptor binding site sequence revealed the presence of E190, G225, Q226, and G228 amino acid residues, consistent with a binding preference for avian-type α2,3-linked sialic acid receptors [8,41]. This suggests limited immediate zoonotic potential; however, receptor binding profiles alone do not fully predict host range. Notably, we identified multiple mutations across the H7N6 genome that are predicted to influence host specificity, receptor binding mechanisms, and potential N-glycosylation sites, which may modulate viral entry, immune evasion, or tissue tropism. Although the well-characterized PB2-E627K mutation, associated with enhanced replication in mammalian cells, is absent, other mutations in polymerase or accessory proteins may still contribute to partial mammalian adaptation or increased virulence [42]. The functional consequences of the mutations detected in the HA receptor binding site require further investigation through structural proteomic approaches to fully understand their potential impacts on host specificity. These observations exemplify the evolutionary plasticity of H5 and H7 LPAI viruses, highlighting the importance of continued surveillance and molecular characterization efforts to detect early signs of pathogenicity shifts in domestic poultry.

Previous research programs and genomic characterization efforts focusing on endemic LPAI strains in New Zealand waterfowl were able to provide critical information necessary to elucidate the origin and emergence of the H7N6 HPAI virus [21]. The availability of genomic data from LPAI strains isolated and sequenced in New Zealand provided the necessary background to rule out an exotic disease incursion event and allowed us to gain a quick understanding of the evolutionary dynamics of the virus. However, given the majority of AIV research in New Zealand is focused on wild waterfowl, there are very limited genomic data available for AIVs detected in host species beyond wild waterfowl in New Zealand [21]. The surveillance data available in poultry is restricted to serological testing for H5 and H7 AIVs [43]. Increased molecular-based monitoring efforts in this space are crucial to improving our understanding of any emergent LPAI viruses within domestic or commercial poultry farms in New Zealand, and would permit the timely detection of other HPAI infection events, including H5N1 HPAI viruses of the subclade 2.3.4.4b [44,45]. Whilst H5N1 HPAI viruses have, to date, never been detected in New Zealand, the emergence of H5N1 HPAI subclade 2.3.4.4b in the Antarctic Peninsula represents an increased biosecurity risk to New Zealand and its surrounding territories, including offshore and subantarctic islands [46,47]. Given the diverse range of avian species found on New Zealand’s offshore and subantarctic islands, and due to the evolving risk of H5N1 HPAI viruses to New Zealand, continued AIV surveillance efforts surrounding aquatic avian species in New Zealand is warranted.

Due to their significant impacts on the poultry industry, and the potential public health implications, AIVs are widely considered to be of high economic importance. The detection of the first case of HPAI in New Zealand was a significant epidemiological event which demonstrated the importance of the research efforts conducted to date, effective preparedness and response planning, and our national capabilities for rapid molecular and genomic diagnostics. Collectively, the surveillance, preparedness, and response efforts conducted by the New Zealand Ministry for Primary Industries enabled the rapid eradication of the H7N6 HPAI virus reported here.

## 4. Materials and Methods

### 4.1. Sample Collection and Nucleic Acid Extraction

Following a mass mortality event on a free-range poultry farm, 31 oropharyngeal swabs and 4 pooled tissue samples (n = 35) were collected post-mortem. No samples were derived from live, symptomatic poultry. Each pooled tissue sample comprised heart, kidney and liver tissue obtained from a single bird. An additional 2 oropharyngeal swabs and 2 cloacal swabs (n = 4) were collected from mallard ducks roaming near the affected free-range poultry farm. All samples underwent total nucleic acid extraction using the MagMAX™ Core nucleic acid purification kit (Applied Biosystems, Waltham, MA, USA) on a KingFisher™ Flex purification system (Thermo Fisher Scientific, Waltham, MA, USA). A total of 34 samples collected from the poultry farm returned positive RT-qPCR results for influenza A [48]. The RT-qPCR used for the initial diagnosis of influenza A targeted a conserved region of the matrix gene, as described in Spackman et al. (2003) [48]. Thermal cycling was performed according to the published method on a CFX Opus qPCR system (Bio-Rad, Hercules, CA, USA). In addition, 17 of the 34 samples returned positive for influenza subtype H7 by RT-qPCR [49]. Thermal cycling was performed according to the published method on a CFX Opus qPCR system (Bio-Rad). For the samples collected from mallard ducks in close proximity to the free-range farm, 1 of the 4 samples returned a positive result for influenza A, which subsequently produced a negative result for H5 or H7 by RT-qPCR, indicating the detection of a non-H5/H7 strain of LPAI.

### 4.2. Whole Viral Genome Amplification and Sequencing

Influenza A-positive samples with Ct values < 30 (n = 18 for the poultry farm, n = 1 for the wild duck) were selected for whole viral genome amplification by RT-PCR, using previously published primers and the SuperScript™ III One-Step RT-PCR system with Platinum™ *Taq* DNA polymerase (Thermo Fisher Scientific) [50]. Thermal cycling was performed using previously published primers and PCR parameters (Method B) on a Veriti 96-well thermal cycler (Applied Biosystems) [50]. Amplified viral genomes were purified using the ExoSAP-IT™ Express PCR product cleanup reagent (Thermo Fisher Scientific) according to the manufacturer’s protocol.

Purified viral genome amplicons were prepared for whole-genome sequencing using the Rapid Barcoding Kit 96 V14 (SQK-RBK114.96, Oxford Nanopore Technologies, Oxford, UK) according to the manufacturer’s protocol for amplicon sequencing. Libraries were pooled, then loaded onto a PromethION flow cell for sequencing using a P2 Solo instrument (Oxford Nanopore Technologies). Sequencing was initiated in MinKNOW v24.06.16 for 24 h, and basecalling was performed using Dorado v7.4.14 with a high accuracy basecalling algorithm. A minimum quality threshold of Q9 was implemented to remove low quality reads. Barcoded samples were automatically demultiplexed in MinKNOW, with raw demultiplexed reads periodically binned into FASTQ files for real-time analysis.

### 4.3. Genome Assembly

Raw sequence reads produced by the sequencing instrument were compiled into one FASTQ file per sample, which was then used for subsequent analysis. Reads from each sample were mapped against a non-redundant set of sequences from the GISAID EpiFlu™ database using Minimap2 v2.28 with the lr:hq model [51]. The target segments with the best mapping rates were identified and compiled into a template genome, then used in a second mapping run to generate sorted BAM files. The BAM files were then used to generate consensus sequences using SAMtools with default parameters [52]. The resulting genomes were corrected using Medaka v1.11.1 (Oxford Nanopore Technologies), followed by manual curation to identify the correct reading frame for each segment, and polishing to remove terminal N sequences.

### 4.4. Avian Influenza Subtype Analysis

Avian influenza A subtype was initially determined in real-time by comparing k-mer profiles of sequenced reads against a non-redundant subset of haemagglutinin (HA) and neuraminidase (NA) gene segments obtained from the GISAID EpiFlu™ database using Flui, an automated subtyping tool developed in collaboration with Dragonfly Data Science, Wellington, New Zealand (https://github.com/dragonfly-science/flui, accessed on 22 August 2025). Subtypes were then confirmed by examining the mapping rates of sequenced reads, using Minimap2 v2.28 with the same non-redundant subset of HA and NA sequences used in the first round of mapping as a reference.

### 4.5. Phylogenetic Analysis

To understand the phylogenetic context of the virus, closely related genomes were identified by performing NCBI BLASTn searches against the AIV genomes present in both the NCBI GenBank and GISAID EpiFlu™ databases, and by aligning the sequenced genome to various LPAI genomes sequenced at the New Zealand Ministry for Primary Industries Animal Health Laboratory. Prior to phylogenetic analysis, an evolutionary model selection test was performed using Molecular Evolutionary Genetics Analysis (MEGA) v11.0.13. Considering the calculated Bayesian Information Criterion (BIC), Akaike Information Criterion corrected (AICc), and log-Maximum Likelihood (lnL) values, the Tamura-Nei (1993) substitution model with allowance for evolutionary invariability was appropriate to model parsimony with a strong fit (see Supplementary Table S2 for model selection test results). Following optimal model selection, phylogenetic trees were constructed using MEGA v11.0.13, utilizing the Maximum Likelihood statistical method with a bootstrap analysis comprising 1000 replicates [25].

### 4.6. Mutation Analysis and Protein Structure Modelling

The genome assembly was uploaded to FluSurver (https://flusurver.bii.a-star.edu.sg, accessed on 22 August 2025) to identify candidate mutations predicted to have significant phenotypic or functional impacts when compared to reference AIV genomes. For each mutation, a level of significance was automatically assigned by FluSurver based on previous reports of similar mutational events in the literature. Candidate mutations of interest were interrogated and individually curated to determine their predicted functional effects. Haemagglutinin protein structure modelling was performed using SWISS-MODEL [36].

## Figures and Tables

**Table 1:** Genome assembly statistics for the H4N6 AIV genome detected in a wild mallard duck near the H7N6 HPAI poultry farm.

Segment	Length (bp)	GenBank ID
1—PB2	2277	PX270996
2—PB1	2271	PX270997
3—PA	2209	PX270998
4—HA	1695	PX270999
5—NP	1494	PX271000
6—NA	1446	PX271001
7—MP	1002	PX271002
8—NS	849	PX271003

**Table 2:** Pairwise comparisons of the H7N6 HPAI genome with other AIVs available in NCBI GenBank. Top two NCBI BLAST hits are shown for each segment.

Segment	Viruses with Greatest Homology	Pairwise ID (%)	GenBank ID
1—PB2	A/Mallard/NZ/W24_2774/2024 (H4N6)	99.1	PX270996.1
	A/Mallard/NZ/W24_6A_c300/2024 (H10N7)	97.7	PX312466.1
2—PB1	A/Mallard/NZ/W24_4A_c39/2024 (H3N8)	96.7	PX271014.1
	A/Red knot/Firth of Thames/1/2024 (H1N9)	94.9	PQ358077.1
3—PA	A/Mallard/NZ/W24_2774/2024 (H4N6)	99.2	PX270998.1
	A/Red knot/Firth of Thames/1/2024 (H1N9)	98.4	PQ358078.1
4—HA	A/Mallard/NZ/W17_18_201/2017 (H7N7)	94.7	PX271007.1
	A/Mallard/NZ/15.880.80.121/2015 (H7N7)	94.1	MH276960.1
5—NP	A/Mallard/NZ/W23_6_308/2023 (H4N6)	98.9	PX271025.1
	A/Mallard/NZ/1365-350/2005 (H6N9)	94.5	CY077587.1
6—NA	A/Mallard/NZ/W24_2774/2024 (H4N6)	98.8	PX271001.1
	A/Mallard/NZ/W23_6_308/2023 (H4N6)	98.7	PX271026.1
7—MP	A/Red knot/Firth of Thames/1/2024 (H1N9)	98.8	PQ358082.1
	A/Mallard/NZ/479-8/2005 (H6N2)	96.0	CY039368.2
8—NS	A/Mallard/NZ/W24_4A_c39/2024 (H3N8)	98.3	PX271020.1
	A/Mallard/NZ/449-68/2004 (H1N2)	96.0	CY077517.1

**Table 3:** Mutations detected in the HPAI H7N6 genome which are predicted to be associated with host specificity shift and/or host cell receptor binding, virulence, or affect the HA receptor binding site. Mutation analysis was performed in GISAID using FluSurver. All mutational events were annotated using H7 numbering. A full list of detected mutations is provided in Supplementary Table S1.

Sequence	Mutation	Predicted Effects and/or Structural Interactions	Reference
1|PB2|A/Chicken/NZ/W24_2595/2024(H7N6)	I292V	Host specificity shift	[27]
4|HA|A/Chicken/NZ/W24_2595/2024(H7N6)	L162V	Antigenic drift, escape mutant, host specificity shift, host cell receptor binding, binding small ligand(s), antibody recognition sites	[28,29]
	N164T	Virulence, antigenic drift, escape mutant, viral oligomerization, antibody recognition sites, binding small ligand(s)	[30]
	T165S	Virulence, antigenic drift, escape mutant, viral oligomerization, antibody recognition sites, binding small ligand(s)	[31]
	G195A	Virulence, antigenic drift, escape mutant, host specificity shift, host cell receptor binding, binding small ligand(s), viral oligomerization, antibody recognition sites	[28,32]
	Q231L	Virulence, viral oligomerization, binding small ligand(s), host cell receptor binding, antibody recognition sites	[33]
	S236P	Host specificity shift, viral oligomerization, binding small ligand(s), antibody recognition sites, host cell receptor binding	[34]

## Data Availability

The H4N6 viral genome sequences generated in this study were deposited into NCBI GenBank under accession numbers provided in Table 1, and in GISAID under the reference number EPI_ISL_20152005. The H7N6 HPAI viral genome sequences were deposited into NCBI GenBank and GISAID according to McCulley et al., 2025 [23].

## Supplementary Materials

The following supporting information can be downloaded at: https://www.mdpi.com/article/10.3390/ijms262110623/s1.

## Appendix A

*Phylogenetic analysis of the internal genes PB2, PB1, PA, NP, MP, and NS*
